# Biological current source imaging method based on acoustoelectric effect: A systematic review

**DOI:** 10.3389/fnins.2022.807376

**Published:** 2022-07-18

**Authors:** Hao Zhang, Minpeng Xu, Miao Liu, Xizi Song, Feng He, Shanguang Chen, Dong Ming

**Affiliations:** ^1^Department of Biomedical Engineering, College of Precision Instruments and Optoelectronics Engineering, Tianjin University, Tianjin, China; ^2^Tianjin Key Laboratory of Brain Science and Neural Engineering, Academy of Medical Engineering and Translational Medicine, Tianjin International Joint Research Centre for Neural Engineering, Tianjin University, Tianjin, China; ^3^National Key Laboratory of Human Factors Engineering, China Astronaut Research and Training Center, Beijing, China

**Keywords:** acoustoelectric effect, current source imaging, focused ultrasound, spatiotemporal resolution, non-invasive neuroimaging

## Abstract

Neuroimaging can help reveal the spatial and temporal diversity of neural activity, which is of utmost importance for understanding the brain. However, conventional non-invasive neuroimaging methods do not have the advantage of high temporal and spatial resolution, which greatly hinders clinical and basic research. The acoustoelectric (AE) effect is a fundamental physical phenomenon based on the change of dielectric conductivity that has recently received much attention in the field of biomedical imaging. Based on the AE effect, a new imaging method for the biological current source has been proposed, combining the advantages of high temporal resolution of electrical measurements and high spatial resolution of focused ultrasound. This paper first describes the mechanism of the AE effect and the principle of the current source imaging method based on the AE effect. The second part summarizes the research progress of this current source imaging method in brain neurons, guided brain therapy, and heart. Finally, we discuss the problems and future directions of this biological current source imaging method. This review explores the relevant research literature and provides an informative reference for this potential non-invasive neuroimaging method.

## Introduction

**Figure 1:**
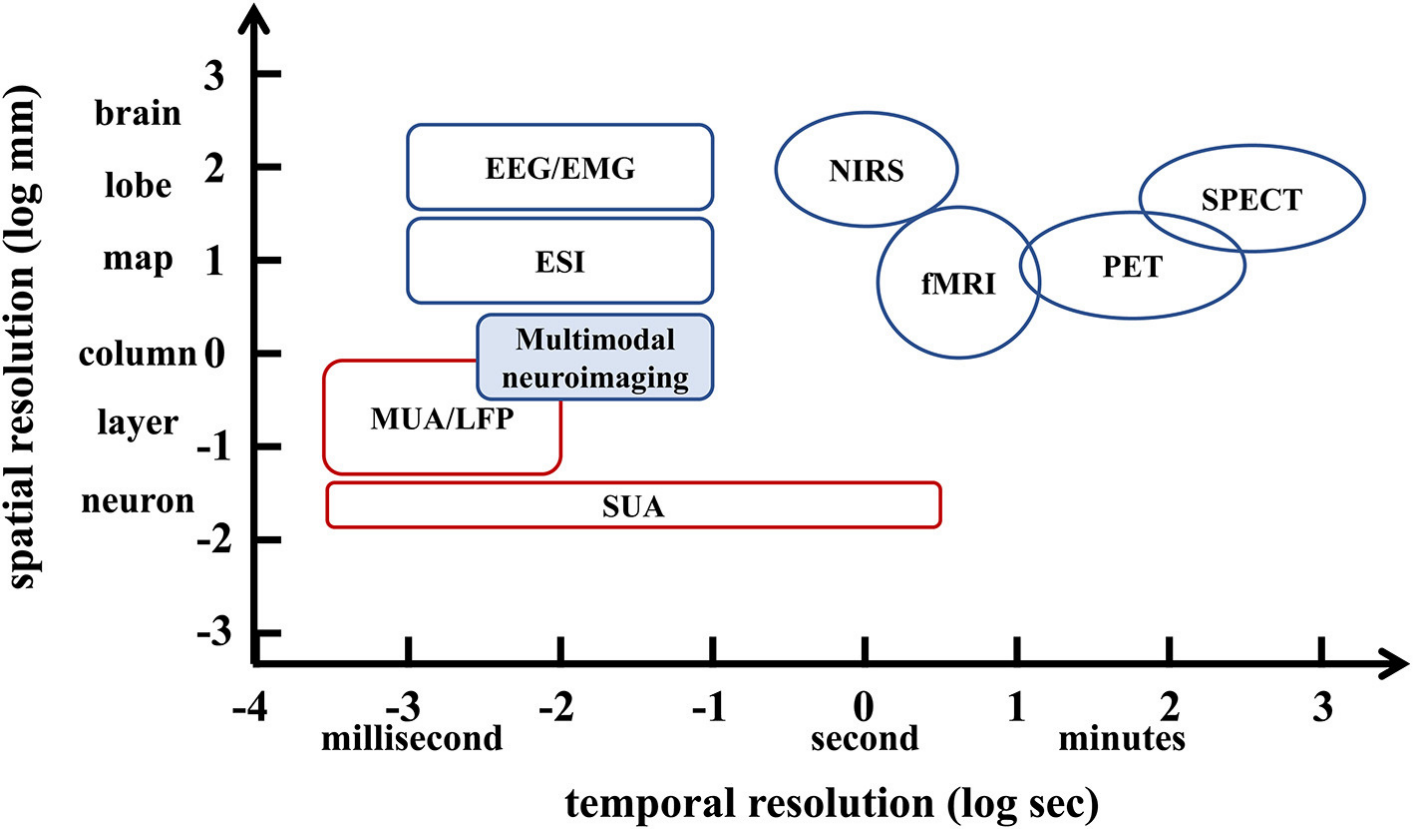
Schematic diagram of the spatiotemporal resolution range of non-invasive imaging techniques (blue) and invasive imaging techniques (red) in the brain.

Imaging the dynamics of normal or abnormal brain activity is important for understanding the brain. However, traditional clinical functional brain imaging techniques have their own drawbacks (Xue et al., 2010). Specifically, invasive neural recordings, such as single-unit activity (SUA), multi-unit activity (MUA), local field potentials (LFP), and electrocorticography (ECoG), have relatively high spatial and temporal resolution. However, they require surgical insertion of electrodes into the brain (Witte et al., 2007; He et al., 2011), which is risky. Non-invasive imaging techniques such as functional magnetic resonance imaging (fMRI), magnetoencephalography (MEG) and electroencephalography (EEG) are convenient and do not cause any harm to the patient. However, the second-level temporal resolution of 3T fMRI, which is commonly used for cognitive neuroscience research and clinical applications, is difficult to capture neuronal activity. That is, the low temporal resolution of fMRI is not sufficient for timely imaging of brain function, and it limits patients with metal implanted devices. MEG/EEG provides high temporal resolution for capturing brain dynamics, but the volume conductor of brain tissue results in low spatial resolution. Here, the temporal resolution (X-axis) and spatial resolution (Y-axis) of current clinical functional brain imaging techniques are shown in Figure 1. With safety in mind, it is difficult for conventional functional brain imaging to have both high temporal resolution and high spatial resolution.

**Figure 2:**
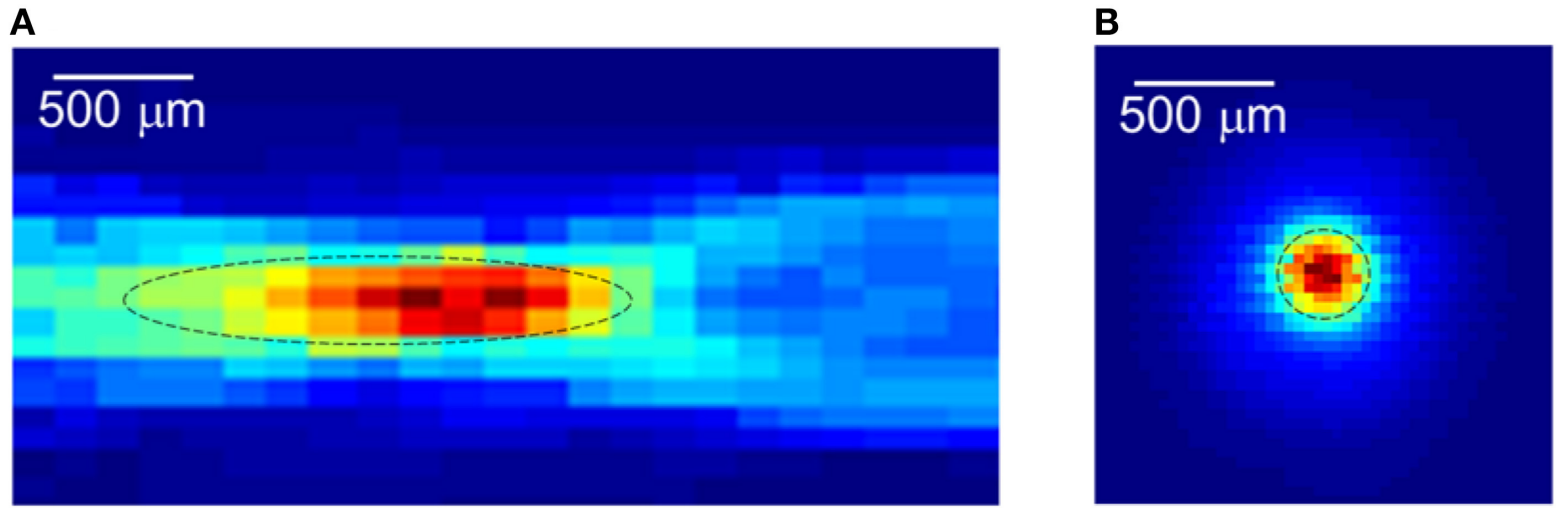
The focused ultrasound focal spot size. **(A)** Lateral focal region. **(B)** Axial focal region. Reprinted with permission from (Evgenii et al., 2019).

Focused ultrasound (FUS) offers the advantages of rapid availability and absence of radiation, while providing real-time focus and high spatial resolution (Figure 2). Based on the acoustic intensity of the focal region, FUS can be divided into high-intensity focused ultrasound (HIFU) and low-intensity focused ultrasound (LIFU). Specifically, HIFU is used to ablate diseased tissue through thermal effects, but it also carries the risk of thermal damage to surrounding normal tissue (Dalecki, 2004; Hynynen and Clement, 2007; O'brien, 2007; Thomas et al., 2020; Ar et al., 2021). In contrast to HIFU, LIFU produces biological effects without thermal damage, and most studies have concluded that LIFU is a safe and effective neuromodulation technique (Dinno et al., 1989; Hynynen et al., 2004; Tyler et al., 2008; Tufail et al., 2010; Seung-Schik et al., 2011; Blackmore et al., 2019; Darrow, 2019).

A literature search was conducted in Web of Science encompassing the studies published before April 1, 2022. The keywords used in the search were “ultrasound” and “acoustoelectric effect,” and literature unrelated to the topic (some literature related to the acoustoelectric conversion of ultrasound transducers) was excluded. Specifically, the AE effect is a fundamental physical phenomenon based on the change of dielectric conductivity. In recent years, AE effect-based biological current source imaging techniques have received a lot of attention in the field of medical imaging. In detail, FUS is used to selectively probe a highly concentrated region of interest (ROI) of the brain to record the electrical signals generated by the neurons in that region. By decoding the AE signals generated in the ROI, the captured information can be used for current source imaging. This imaging method combines the advantages of high spatial resolution of FUS with the advantages of high temporal resolution of electrical signal transmission, and it promises to provide unprecedented high spatiotemporal resolution for non-invasive neuroimaging.

In this review, we first introduce the basic mechanisms of the AE effect and the principles of current source imaging based on the AE effect. In the next sections, the progress of this current source imaging approach in brain neurons, brain-guided therapy, and cardiac research is explored in detail. The final section discusses some potential challenges and future directions of this biological current source imaging approach. This paper provides a review of related research and aims to provide an informative reference for researchers in the field of biomedical imaging.

## Mathematical principle

### AE effect

The acoustoelectric (AE) effect was first discovered by Fox in 1946. He showed that ultrasound can periodically change the electrical conductivity of saline solutions through pressure (Fox et al., 1946). Researchers have spent decades to study the mechanism of the AE effect in depth. Indeed, ultrasound mainly changes physical and chemical properties such as molar concentration, ion dissociation equilibrium, and ion mobility, accounting for 47, 35, and 18% of the conductivity change, respectively (Jossinet et al., 1998, 1999; Wen and Balaban, 1998). Shortly thereafter, the potential of AE effects for biomedical imaging systems was noticed. Specifically, the change in resistivity ρ due to FUS is generally expressed as


(1) $$\begin{array}{l} \frac{\Delta \rho }{\rho_{{\;}_{0}}}=K_{I}\Delta P, \end{array}$$

where Δρ is the change in resistivity, ρ__0__ is the intrinsic resistivity, Δ*P* is the acoustic pressure, and *K*_*I*_ is the interaction constant, which in a 0.9% NaCl solution is of the order of 10^−9^*Pa*^−1^ (Yang et al., 2011; Li et al., 2012). It leads to an increase in conductivity when the pressure drops and a decrease in conductivity when the pressure rises.

### Principle of current source imaging based on the AE effect

#### Mathematical theory

**Figure 3:**
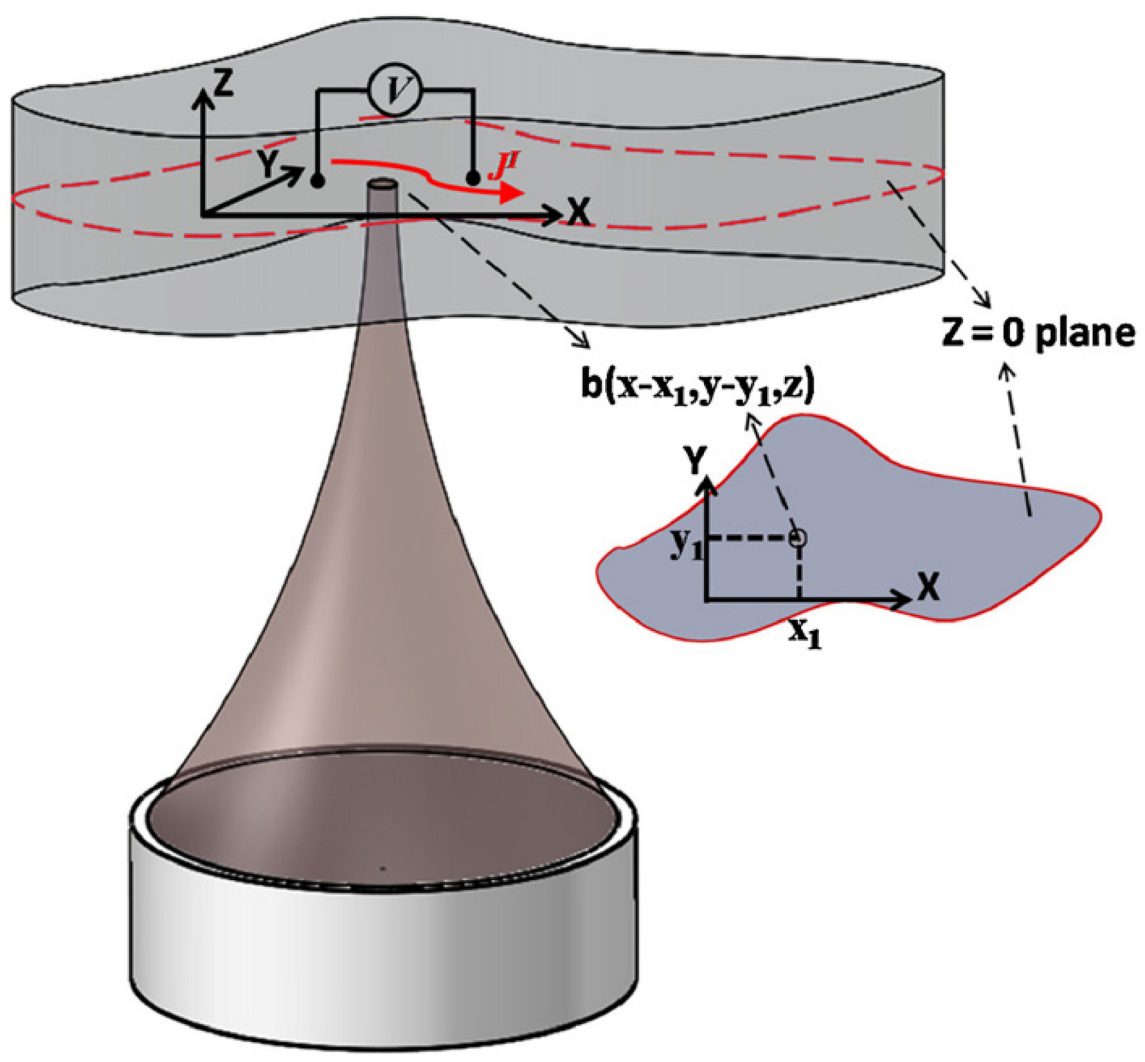
Illustration of the principle of current source imaging based on acoustoelectric effect. It is assumed that the ultrasound focus, recording electrode lead and bio-current are all in the same plane Z=0. A pair of leads is measured for injected voltage (low frequency) and induced acoustoelectric voltage (high frequency). Ultrasound scans the target area point by point and acquires the acoustoelectric signal for 2D current source imaging. Reprinted with permission from (Li et al., 2012).

The principle of AE effect-based current source imaging is schematically illustrated in Figure 3. The FUS transducer located at the bottom of the imaging target periodically sends ultrasound pulses to the conducting object. The ultrasound scan is performed in such a way that the focus of the ultrasound transducer scans the entire volume of the imaged object while collecting the corresponding electrical signals associated with the periodic ultrasound pulses at each scan position. A detailed derivation of the AE effect-based current source imaging formulation can be found in Olafsson et al. (2006, 2007b), Witte et al. (2006), and Li et al. (2011). In general, the electrical signal collected by the electrodes can be described by Eq. 2.


(2) $$\begin{array}{l} V_{i}=∭\rho \left(\tilde{J_{i}^{L}}\cdot J^{I}\right)dxdydz, \end{array}$$

where *J*^*I*^ = *J*^*I*^(*x, y, z*) is the distributed current source, $\tilde{J_{i}^{L}}=\tilde{J_{i}^{L}}\left(x,y,z\right)$ is the lead field of lead *i*, and ρ = ρ(*x, y, z*) is the resistivity. Then, ρ can be expressed as ρ = ρ__0__−Δρ, and Eq. 1 can be transformed into Δρ = *K*_*I*_ρ__0__Δ*P*. Therefore, Eq. 2 can be rewritten


(3) $$\begin{array}{l} V_{i}=∭\left(\tilde{J_{i}^{L}}\cdot J^{I}\right)\rho_{{\;}_{0}}dxdydz+∭\left(\tilde{J_{i}^{L}}\cdot J^{I}\right)\\ \left(-K_{I}\rho_{{\;}_{0}}\Delta P\right)dxdydz. \end{array}$$

The first of these terms is called $V_{i}^{LF}$ and represents the low-frequency content of *V*_*i*_. The second term is called $V_{i}^{AE}$, which represents the high-frequency AE signal. the AE signal is easily filtered out from other signals in living tissue, such as in brain tissue or myocardial tissue. In $V_{i}^{AE}$, we extend the ultrasonic pressure factor to its subcomponents, thus


(4) $$\begin{array}{l} \Delta P\left(x,y,z,t\right)=P_{0}b\left(x,y,z\right)a\left(t-z/c\right) \end{array}$$

where *P*_0_ is the amplitude of the pressure pulse, *b*(*x, y, z*) is the ultrasound beam pattern, *a*(*t*) is the pulse waveform, and *c* is the speed of sound. Inserting (4) into (3), we rewrite $V_{i}^{AE}$ as


(5) $$\begin{array}{l} V_{i}^{AE}\left(x_{1},y_{1},t\right)=-K_{I}∭\left(\tilde{J_{i}^{L}}\cdot J^{I}\right)\rho_{{\;}_{0}}b\left(x-x_{1},y-y_{1},z\right)\\ P_{0}a\left(t-z/c\right)dxdydz \end{array}$$

where $V_{i}^{AE}\left(x_{1},y_{1},t\right)$ represents the voltage sequence of the AE signal during ultrasound irradiation (*x*_1_, *y*_1_). The target tissue is electronically scanned at high speed using ultrasonic pulses, and the voltage signal at each point of ultrasonic irradiation is collected in real time, and this voltage signal is used as raw data. The raw data is first pre-processed by down-sampling and removing industrial frequency interference. Then high-frequency band-pass filtering is performed to retain only the high-frequency signal at the ultrasound frequency (ultrasound center frequency or pulse repetition frequency, PRF). Finally, the high-frequency signals obtained under different experimental conditions are decoded accordingly to obtain the AE signals at each point, and the collection of AE signals at each point forms a spatial map of the current distribution.

#### Signal characteristics

It is well known that FUS can focus acoustic power to a very small area; that is, it can use the focused mechanical energy to provide a high spatial resolution of ROI targeting. At the same time, the AE signal is only proportional to the local lead field in the ultrasound focal zone, not to the whole lead field, which has been confirmed experimentally and theoretically (Olafsson et al., 2008b; Li et al., 2012). The focal zone of an ultrasound transducer depends on many factors, including its center frequency and geometry. In AE imaging studies, considering the process difficulty of the transducer and the imaging accuracy requirements, a millimeter elliptical ultrasonic focal zone is generally used. This is why the AE signal can be used as a spatial marker with a spatial resolution of millimeter-scale.

The main challenge with the AE signal applied to imaging human tissue current sources is that it is very low in magnitude. It is only a few tens of microvolts and is more likely to be drowned out by noise (Helgason and Gunnlaugsdottir, 2015). Several efforts have been made to improve the AE signal-to-noise ratio (SNR), such as optimizing the ultrasound drive system and signal acquisition circuitry. Qin described the performance of a custom 0.6 MHz 2D ultrasound array designed for transcranial acoustoelectric brain imaging (ABI) through the adult skull. He found that the sensitivity was greatly improved with frequency-encoded ultrasound excitation, which in itself could improve the SNR by more than 10 dB without sacrificing spatial resolution. Moreover, AE signals could be detected at depths >40 mm from the skull surface (Qin et al., 2017). In the acquisition circuit section, previous studies have shown that the addition and subtraction of the AE signal acquired by two Wheatstone bridge circuits are independent components, regardless of the bridge circuit's location. The two complementary bridge circuits reduce common-mode noise and allow AE signal detection with only two pairs of recording electrodes and one pair of stimulation lines. In all experiments, the bridge circuit showed a significant improvement in SNR compared to the conventional circuit (Wang et al., 2016a).

Researchers have also investigated in-depth the effect of ultrasonic polarity on the AE signal. First, since bipolar or balanced oscillations tend to average any local phenomenon, leading to a loss of AE signal, the conventional inverse method in (Yang et al., 2011) becomes inapplicable and susceptible to small measurement noise. There are two possible approaches to solve this problem, and the first solution is to improve the bipolar pulsed ultrasonic current imaging method. The Werner deconvolution-based ultrasound current imaging scheme helps to reconstruct high-quality current distributions (Renhuan et al., 2013). The second approach is to use unipolar ultrasound pulses. For this purpose, researchers first used unipolar ultrasound pulses to quantitatively assess ultrasound-induced resistance changes in saline solutions (Lavandier et al., 2000). Conventional sine waves have two half-cycles with opposite polarity, which produce opposite conductivity changes over a distance of one wavelength, so their effects tend to neutralize each other, which directly reduces the amplitude of the AE signal. In contrast, unipolar pulses do not have this problem, and the results also prove that this excitation method gives a more general and accurate inverse solution. However, unipolar pressure pulses are very demanding on the material and structure of the ultrasonic transducer and are prone to waveform distortion problems.

It can be foreseen that with the advancement of ultrasound transducer devices, the second method may gradually replace the former for more highly accurate current source reconstruction. Although the AE effect has a short history of application in biomedical imaging, the potential of current source imaging has attracted extensive attention from researchers.

## Research progress

Worldwide, there are approximately 50 million patients with epilepsy. Most patients require neurosurgical treatment, which requires prior knowledge of the area of abnormal neural discharge (Sood and Chugani, 2006; Engel, 2010; Acharya et al., 2013; He and Bin, 2016). One obstacle to the wider use of epilepsy surgery is the difficulty of identifying the region of seizures by non-invasive means. Traditional methods require painstaking and prolonged invasive EEG. In addition to this, arrhythmic activity is an important part of the ongoing spontaneous brain activity (Zempel et al., 2012). More than 30,000 arrhythmia treatment procedures are performed worldwide each year (Mickelsen et al., 2005; Kumar et al., 2020). This usually includes cardiac catheter ablation and pacemaker implantation interventions (Morady, 2004; Olafsson et al., 2008b; Li et al., 2012).

Despite the importance of current source imaging for brain and heart treatment, current imaging methods are difficult to have both non-invasive and high spatiotemporal resolution. In recent years, hybrid current source imaging techniques, represented by AE effect-based current source imaging, have been widely pursued (Witte et al., 2007; Olafsson et al., 2008b, 2009; Wang et al., 2011; Li et al., 2012; Qin et al., 2012b, 2015; Renhuan et al., 2013; Wang and Witte, 2014). These techniques not only allow imaging of currents injected from outside and have the promise of imaging real currents in tissue (see Table 1 for details). Next, we will describe the progress of such current source imaging in three aspects. (1) Current source imaging methods for the brain neurons; (2) current source imaging methods for guiding brain therapy; and (3) current source imaging methods for the heart.

**Table 1:** The main research progress of current source imaging based on AE effect.

**Author/year**	**Transducer**	**Frequency** **(center frequency)**	**Experimental objects**	**Main results**
Olafsson et al. (2006)	Single-element	0.54 MHz	Two tin electrodes 0.9% nacl solution	Reconstruction of simulated ECG waveforms from ultrasound-modulated voltage traces.
Olafsson et al. (2007b)	Single-element	7.5 MHz	0.9% nacl solution	The UCSDI positions the current source and current sink within 1 ± 2 mm of the actual location.
Olafsson et al. (2007a)	Single-element (etalon, in)	0.54 MHz PRF:1,600 Hz	Isolated rabbit heart	The first direct measurement of cardiac activation waves using UCSDI.
Olafsson et al. (2008b)	Single-element	7.5 MHz	0.9% nacl solution	Position the current source and current sink within 1 mm of their actual position by UCSDI.
Olafsson et al. (2009)	Single-element (etalon, indianapolis, in)	0.54 MHz PRF:1,600 Hz	Live rabbit heart	The UCSDI has been used for the first time to map biological current in the hearts of living rabbits.
Wang et al. (2011)	Single-element	1 MHz PRF:2,500 Hz	0.9% nacl solution	The FUS beam combined with a single recording electrode and ground reference is sufficient to produce a volumetric image of the time-varying current distribution in a conducting medium.
Yang et al. (2011)	Simulation	2 MHz	Simulated homogeneous and inhomogeneous 0.9% nacl solution	Current source imaging based on monopolar pressure pulses gives a more general and accurate inverse solution.
Qin et al. (2012b)	Single-element	1 MHz 2.25 MHz	0.9% nacl solution	The chirp has a higher sensitivity (3.5 μV/mA) compared to the square pulse excitation (1.6 μV/mA).
Li et al. (2011)	Single-element (panametrics)	1 MHz	Porcine heart tissue cylindrical gel (1.5% agarose and 0.9% nacl)	The sensitivity of UCSDI was 4.7 μV/mA in cylindrical gels (0.9% NaCl) and 3.2 μV/mA in porcine heart tissue.
Qin et al. (2012a)	Single-element (panametrics v394)	1 MHz	Live rabbit heart	Compared with square wave pulses, chirp excitation improves the detection of AE signals by up to 6.1 dB.
Li et al. (2012)	Single-element (panametrics)	1 MHz	Seven cadaver rabbit hearts Divalent salt cuso_4_ solution Monovalent salt nacl solution	In rabbit hearts, K was determined to be 0.041 ± 0.012%/MPa, similar to the measurement of K in saline (0.034 ± 0.003%/MPa).
Wang and Witte (2014)	Single-element	1 MHz PRF:2.5 kHz	0.9% nacl solution	The AE signal is sensitive to the distance from the dipole, but less sensitive to the angle between the detector and the dipole.
Qin et al. (2014)	Single-element (panametrics v389/a392s)	0.5 MHz PRF:2 kHz	Live rabbit heart	The first 3D heart activation map was presented.
Qin et al. (2015)	Single-element (olympus panametrics-ndt v389/a392s)	0.5/1 MHz	Live rabbit heart	For the first time, only one pair of recording electrodes was used to record a 3D cardiac activation map of a live rabbit heart.
Qin et al. (2016)	Single-element (panametrics v389/a392s)	0.5/1 MHz	Human skull replica made of resin Brain phantom (0.9% nacl and 1% agarose gel)	Using a single-element transducer and copper recording wire, the detection threshold for a current source more than 15 mm below the surface of the brain model was <1 mA/cm^2^.
Wang et al. (2016a)	Single-element	2.5 MHz	Turkey slices (cut into long, thin rectangles) 0.9% nacl solution	The AE signal and SNR are stronger in the presence of the bridge circuit compared to the absence of the bridge circuit.
Tseng et al. (2017)	Single-element (olympus ndt a389)	0.5 MHz	Simulation	Compared to short linear frequency modulated pulses (chirp), a non-linear chirp with optimized inverse filtering can improve SNR by >6 dB under certain conditions.
Preston et al. (2017)	Single-element (olympus ndt a392s)	1 MHz	0.9% nacl solution	The AEI is able to accurately determine the polarity, magnitude, and location of the current density in the vicinity of a DBS device placed in saline.
Preston et al. (2018)	Single-element (ndt a392s, olympus, shinjuku, tokyo, japan)	1 MHz PRF:4 kHz	Human skullcap 0.9% nacl solution	The AE signal is 10 dB above the background with a sensitivity of 0.56 ± 0.10 mV/(mA*MPa).
Preston et al. (2019)	Linear array (philips p4-1, 96 elements)	2.5 MHz PRF:8 kHz	Human skull	The AEI can provide non-invasive, high-resolution feedback on current diffusion at directional DBS electrodes.
Zhou et al. (2019)	Single-element (olympus a392s)	1 MHz PRF:100/200/500/1,000 Hz	0.9% nacl solution	The pFU has a modulation mechanism for the source signal at PRF and confirms the feasibility of recovering the source signal from the modulated AE signal.
Zhou et al. (2020b)	Single-element (olympus a392s)	1 MHz PRF:100/200/500/1,000/2,000 Hz	0.9% nacl solution Vivo rat brain	Both the AE signal envelope and the decoded AE signal were significantly correlated with the low-frequency EEG.
Zhou et al. (2020a)	Single-element (olympus a392s)	1 MHz PRF:100/200/500/1,000 Hz	0.9% nacl solution	Multiple sources with different frequencies and amplitudes are not only clearly imaged, but also the corresponding features can be further extracted from the AE signal.
Preston et al. (2020)	Linear array (philips, p4-1, 96 elements)	2.5 MHz PRF:6 kHz	Three adult human skulls 0.9% nacl solution	Adjacent contacts along the length of the leads and within each ring (average radial spacing of 2.10 and 1.34 mm) are identifiable.
Barragan et al. (2020)	Linear array (vantage 64 le, verasonics, kirkland, wa) /matrix array (sonic concepts, bothell, wa)	3 MHz PRF:4 kHz 0.6 MHz	Adult human cadaverskul gel phantom (0.9% nacl and 1% agarose or 5% porcine gelatin in dih_2_0)	This study was able to map current source densities up to 63 mm deep with high spatial resolution and present fast time-varying currents with sub-second accuracy.
Alvarez et al. (2020a)	Matrix array (126 elements, sonic concepts; bothell, wa)	0.6 MHz PRF:4 kHz	Vivo swine cardiac	This study demonstrates for the first time *in vivo* acoustoelectric cardiac imaging in a swine model.
Song et al. (2021)	Single-element (olympus a303s)	1 MHz PRF:80/90 Hz	Vivo rat brain	The first SSVEP measurement with millimeter-level spatial resolution in live rats was achieved by ABI.
Zhang et al. (2022)	Single-element (olympus a392s)	1 MHz PRF:662 Hz	Brain tissue phantom fresh porcine brain tissue	The study locates and decodes dipole signals with high accuracy from both experimental and simulation aspects.

### Current source imaging methods for brain neurons

**Figure 4:**
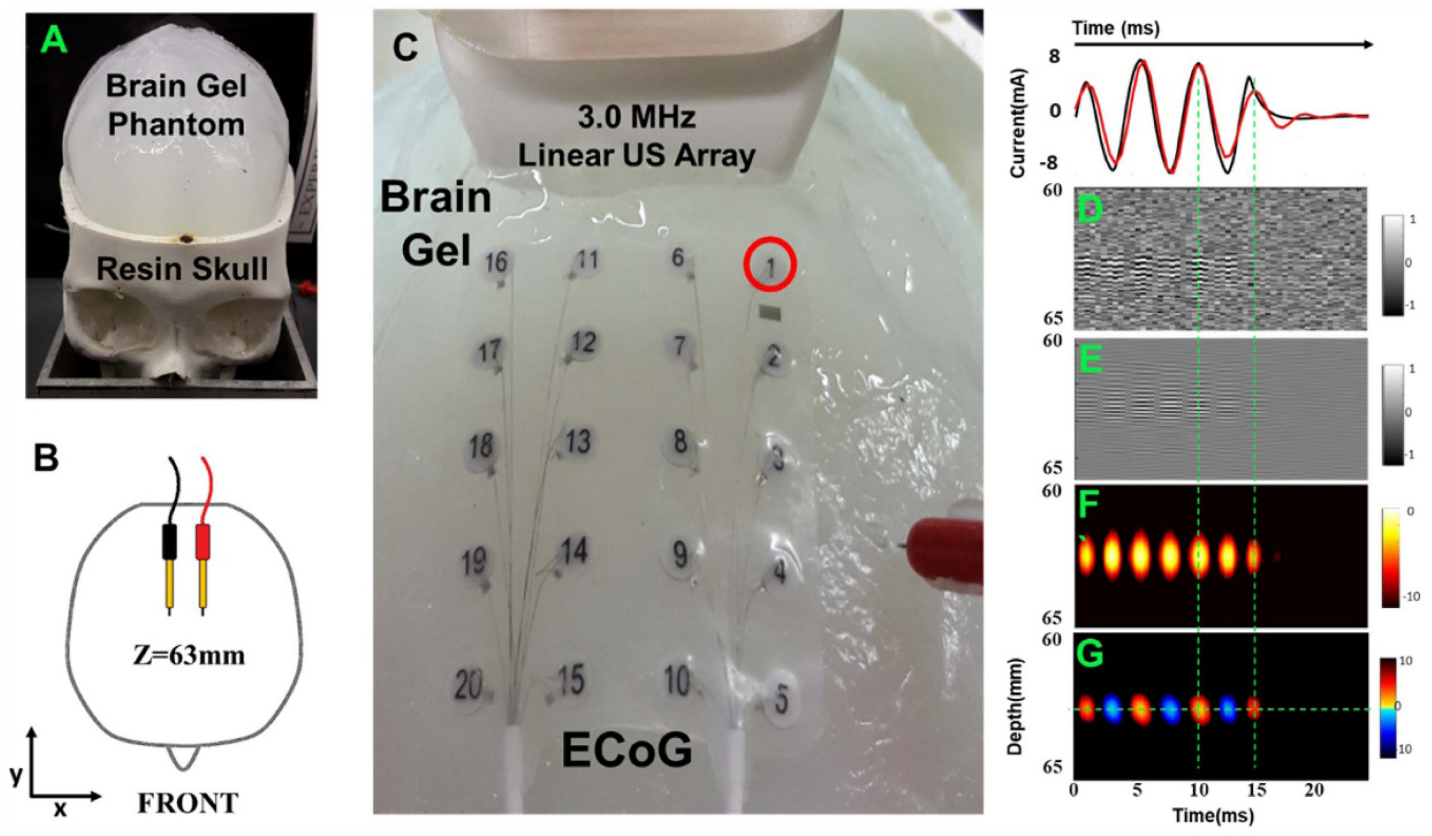
Acoustoelectric M-mode images of a deep dipole with the open-skull model. **(A)** A 3D-printed resin skull served as the frame with the skullcap removed. **(B)** A pair of dipoles. **(C)** Close-up view of setup with the 3 MHz linear array, 20-electrode array (on brain gel surface), and needle reference. **(D)** The raw unfiltered image. **(E)** Bandpass filtered image. **(F)** Envelope image. **(G)** The signed envelope image. Reprinted with permission from (Barragan et al., 2020).

As mentioned by Qin et al., current source imaging methods applied to the brain are commonly referred to as ABI (Qin et al., 2016, 2017). This section focuses on the progress of ABI in simulated current detection and rat brain studies. To simulate neural firing, researchers developed a human skull and brain phantom to test and optimize ABI detection of embedded “EEG-like” currents. Because the skull is a strong absorber and disperser of ultrasound and electrical signals (Zhang et al., 2021), in earlier studies, the skull cap was removed and ultrasound waves were delivered directly through the surface of the brain phantom (Qin et al., 2016). Barragan used a head phantom with a real human skull to demonstrate 4D ABI for mapping time-varying monopoles and dipoles at depths >60 mm with detection thresholds <0.5 mA (Figure 4) (Barragan et al., 2020). In 2022, Zhang used FUS irradiation to simulate the dipole of neuronal firing, and the simulation and experimental results showed that the localization and decoding results were highly consistent with the predicted situation (Zhang et al., 2022)).

Meanwhile, Burton demonstrated the feasibility of using ABI to map the physiological activity of the rat hippocampus based on depth and surface recording electrodes (Burton et al., 2018). Zhou proposed a source signal modulation mechanism for ABI with pulsed focused ultrasound (pFU). And they further demonstrated the feasibility of recovering the source signal from the modulated AE signal (Zhou et al., 2019). This encoding mechanism was further validated *in vivo* rat experiments with different PRFs including 500 Hz, 1 kHz, and 2 kHz. Both the AE signal envelope and the decoded AE signal showed a significant correlation with low-frequency EEG (Zhou et al., 2020b). Next, Zhou evaluated and validated multi-source ABI with different current source functions. The multi-sources with different frequencies and amplitudes not only allowed clear imaging but also efficiently extracted the corresponding features from the AE signal. It verified the feasibility of multi-source ABI with different current source functions (Zhou et al., 2020a).

It is important to note that the source signal was modulated using pulsed ultrasound PRF in Song and Zhou's study, which differs from the methods of other researchers. If we strictly follow the mathematical theory, the AE effect and AE signal are defined at the US carrier frequency (MHz). However, whether the signal generated at the PRF is an AE signal in the theoretical sense or not, this has not been rigorously theoretically derived. Perhaps, there is another explanation or mechanism for the measured signal in these papers? Although some progress has been made in imaging and source signal extraction by the signal obtained at the PRF, this measured signal is in urgent need of sufficient theoretical/equation support. In addition, does the measured signal obtained at the PRF lose some critical temporal information or a certain amount of amplitude compared to the conventional AE signal? In other words, the relationship between these two signals and their respective applicability needs to be further investigated.

Recently, Song performed experiments to measure SSVEP in live rats based on ABI. The decoded AE signal has a high amplitude response at the fundamental and harmonic of the visual stimulus frequency. This study achieved the first SSVEP measurement with millimeter spatial resolution in ABI live rats (Song et al., 2021)). The results of phantom experiments and animal experiments demonstrate that ABI is able to localize simulated current sources or firing neurons at high resolution under a variety of conditions, demonstrating the feasibility of ABI for detecting intracranial neuronal firing (Barragan et al., 2020). In the next study, as a transition between mimic brain tissue phantom experiments and *in vivo* brain experiments, we can try to use live brain slices for bio-current source imaging in *in vitro* experiments, which is a must before its application in the clinic.

### Current source imaging methods for guiding brain treatment

In addition to detecting neuronal firing, current source imaging can map the current distribution of invasive therapeutic devices to guide the treatment of neurological disorders. Deep brain stimulation (DBS) can provide more precise electrical stimulation of targeted brain structures in Parkinson's disease, primary tremors, and other neurological disorders. While intraoperative navigation *via* MRI or CT allows placement of DBS leads with near millimeter accuracy, none of the existing modalities provide feedback of current diffusion from the contacts to the brain tissue. Researchers investigated transcranial acoustoelectric imaging (AEI) as a new modality for non-invasive imaging and characterization of currents generated by directed DBS leads.

Preston noted that AEI could accurately determine the polarity, magnitude, and location of the current density near the DBS device placed in saline using stimulation parameters similar to those of the patient with the SNR of 17.1 dB. Also, pulsed-echo (PE) ultrasound was acquired to provide additional information about the spatial coordinates and structure of the DBS without the need for other techniques (Preston et al., 2017). He then inserted DBS electrodes into a colloidal model of the brain inside the human skull and then modulated a linear array of ultrasound transducers to focus near the electrodes. The results showed that the SNR of the detected AE signals ranged from 7 to 16 dB at an injection current of 11 mA and a focusing pressure of 2.04 MPa. AEI can provide non-invasive, high-resolution feedback of current diffusion from a directional DBS electrode (Preston et al., 2019). In addition, Preston constructed monopolar and dipolar models in saline with and without a human skullcap. AEI can accurately position DBS leads to within 0.70 mm, with a detection threshold of 1.75 mA at 1 MPa and a sensitivity of 0.52 ± 0.07 μV/(mA^*^MPa) (Preston et al., 2018). On this basis, he inserted 8-channel directional DBS leads into three adult human skulls immersed in 0.9% NaCl and used a 2.5 MHz linear array to focus near the contacts on the leads. When using a safe ultrasound pressure and an injection current with a peak amplitude of 2 mA, the AEI detected monopolar currents with stimulation pulses as short as 100 μs and a SNR of 10–27 dB (Preston et al., 2020).

This imaging modality could improve the accuracy of placing DBS leads, guide calibration and monitor the long-term performance of DBS for the treatment of neurological disorders. These efforts suggest that AEI may become a revolutionary modality for real-time high-resolution current mapping to monitor, stage, and guide the treatment of epilepsy and other disorders characterized by abnormal rhythms.

### Current source imaging methods for heart

In addition to the wide range of applications in the brain, the advantage of the method to clearly visualize the properties of electrical currents makes it also useful in the treatment of cardiac system diseases. Prior to surgical procedures, the electrical system of the patient's heart should be obtained, usually measured by direct electrophysiological (EP) mapping. However, EP mapping suffers from ionizing radiation, long imaging times, poor spatial resolution, low SNR (Goldberger, 2006; Klemm et al., 2007), and high geometric alignment errors with computed tomography (CT) or magnetic resonance imaging (MRI) (Daccarett et al., 2007; Zhong et al., 2007). Currently, researchers primarily use electro anatomical mapping (EAM) to facilitate the mapping of cardiac activation waves and to identify re-entry currents, which is the gold standard for mapping the heart prior to ablation (Packer, 2005). However, EAM has registration distortion and cannot monitor cardiac activation waves and non-sustained arrhythmias, such as ventricular tachycardia, in real time (Duru, 2002). In other words, conventional methods of cardiac potential conduction mapping suffer from low spatial resolution, low SNR, and long imaging times (He and Wu, 2001). The ultrasound current source density imaging (UCSDI) is a very effective method for mapping cardiac potential conduction (Li et al., 2012), and it has been demonstrated by various techniques (e.g., time-varying dipoles) (Nguyen et al., 2008; Wang et al., 2016b).

As a proof of principle, Olafsson used a 540 kHz ultrasonic transducer focused between two tin electrodes immersed in a sodium chloride solution. Simulated electrocardiogram (ECG) waveforms were successfully reconstructed from ultrasound-modulated voltage traces (Olafsson et al., 2006). Olafsson generated a 2D dipole field in a 0.9% NaCl solution through a pair of electrodes. He sequentially irradiated the entire solution with a 7.5 MHz transducer and further estimated the direction and magnitude of the current field at each point based on AE effect. The correlation coefficient between the UCSDI and the simulated field was 0.9957, allowing the current source and sink to be positioned within 1 ± 2 mm of their true locations (Olafsson et al., 2007b). Similarly, the reconstructed images in the fresh lobster nerve cord were also consistent with AE simulations (Witte et al., 2006).

**Figure 5:**
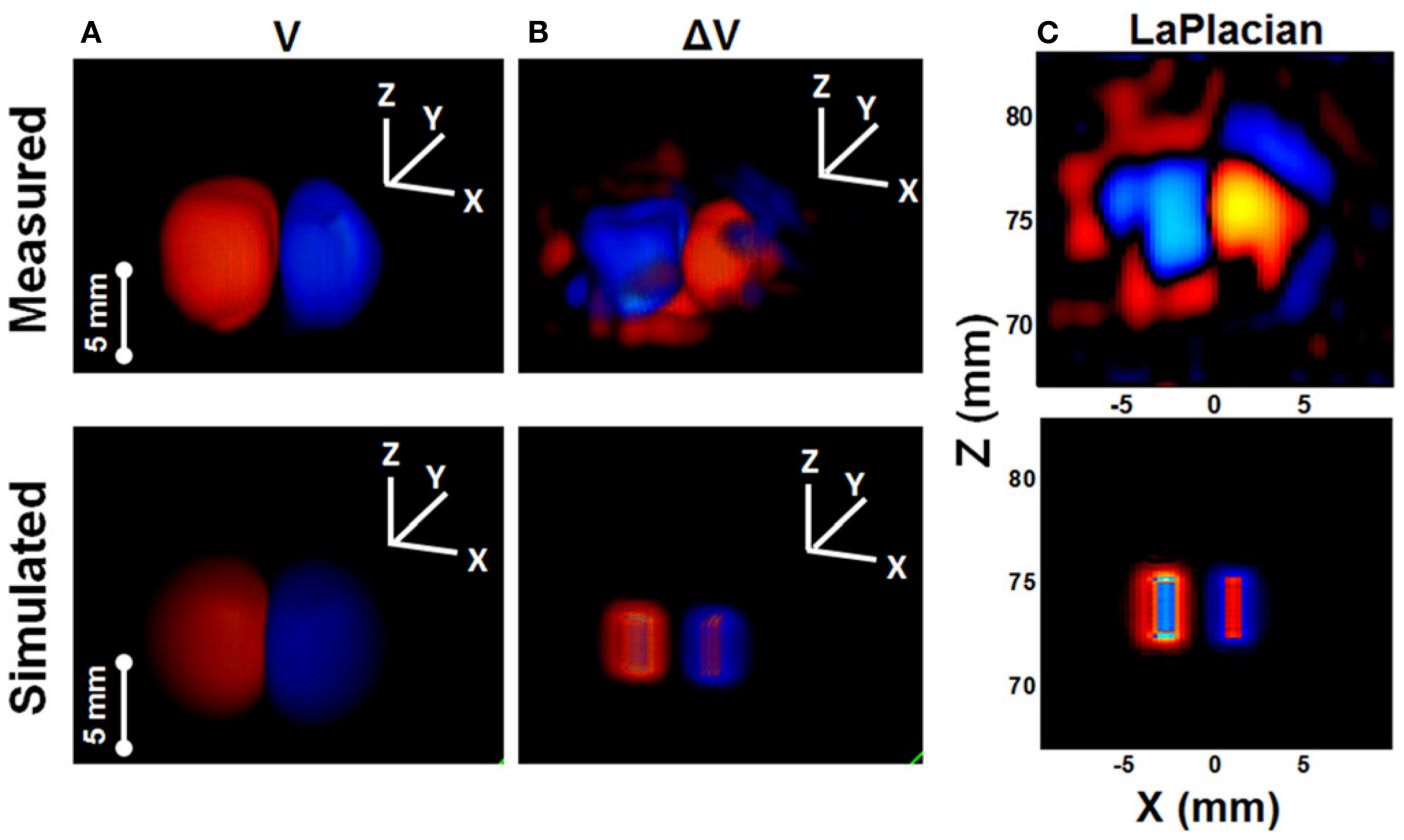
Comparison between measured images (ultrasound current source density imaging, UCSDI) with simulated images (COMSOL Multiphysics) of the current dipole in saline. **(A)** Voltage potential. **(B)** Current source from Laplacian of **(A)**. **(C)** The X-Z plane through the center of Laplacian image. Reprinted with permission from (Wang et al., 2011).

Over the next year, Olafsson scanned an isolated rabbit heart using a 540 kHz transducer, which he perfused with an excitation-contraction decoupler to reduce cardiac motion. A tungsten electrode inserted into the left ventricle allowed simultaneous recording of both high-frequency AE signals and low-frequency ECGs. Its spatiotemporal pattern and propagation velocity were consistent with cardiac activation waves, demonstrating UCSDI as a potential technique for mapping potential cardiac conduction (Olafsson et al., 2007a, 2008a). In addition, he used UCSDI for the first time to map the anatomical potential conduction of the living rabbit heart, which could provide the spatiotemporal distribution of cardiac activation waves (Olafsson et al., 2009). Alvarez later demonstrated current source imaging *in vivo* for the first time in a swine model (Alvarez et al., 2019, 2020a,b). Wang also used a pair of electrodes to generate alternating current distributions in a special imaging chamber filled with 0.9% NaCl solution, and obtained time-lapse volume movies of the alternating current distribution (Figure 5) (Wang et al., 2011).

Next, Li focused on the interface between UCSDI and clinical intracardiac catheters—a key step in translating UCSDI into guided cardiac ablation therapies. The sensitivity of UCSDI binding to the catheter was tested in a model of simulated tissue. The sensitivity with the catheter was 4.7 μV/mA in a cylindrical gel (0.9% NaCl) and 3.2 μV/mA in porcine heart tissue (Li et al., 2011). Qin used a rabbit Langendorff heart preparation to study the effect of electrode configuration and ultrasound frequency on the magnitude of the AE signal and the quality of the UCSDI. The results demonstrate that the AE signal at 0.5 MHz (2.99 μV/MPa) is much stronger than that at 1.0 MHz (0.42 μV/MPa) (Qin et al., 2015).

**Figure 6:**
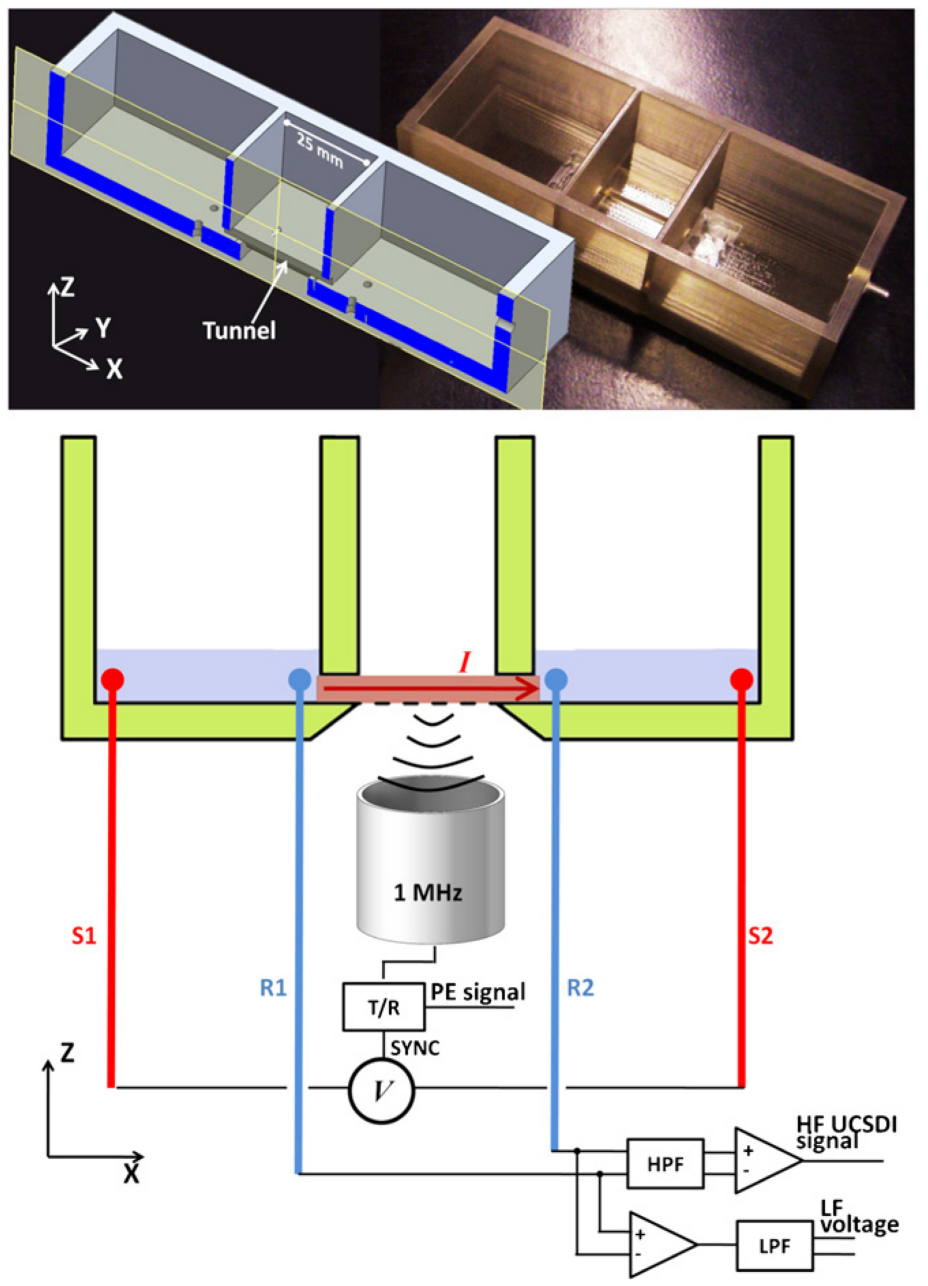
The X-Z cross-sectional view of the design chamber (left) and the actual photograph (right). The chamber has three connected compartments: two side compartments for electrolyte and the middle compartment for placement of the samples. Reprinted with permission from (Li et al., 2012).

In terms of improving the quality of UCSDI, Tseng also investigated the role of pulsed waveforms in amplifying the AE signal and improving the imaging SNR. A non-linear chirp with optimized inverse filtering can improve SNR by >6 dB under certain conditions compared to a short linear frequency modulated pulse (chirp) (Tseng et al., 2017). They also demonstrated that chirp excitation can improve the SNR (Qin et al., 2012a) and sensitivity (Qin et al., 2012b)) of AE signals compared to broadband square wave pulse excitation. In addition to this, they designed a test chamber (Figure 6) and found that the AE interaction constant K was strongly concentration dependent for the divalent salt CuSO4, but not for the monovalent salt NaCl (Li et al., 2012). Subsequently, Berthon's study was based on a 5 MHz linear array of 256-channel ultrasound platforms with simultaneous UCSDI, ultrafast B-mode, and ECG recordings. UCSDI signals from different electrode locations along the myocardial wall showed the possibility of mapping the electrical activation of the whole heart (Berthon et al., 2019).

UCSDI has made some progress in the treatment of cardiac arrhythmias. Its imaging objects have evolved from the initial saline to phantoms, then to isolated animal hearts, and finally to the current live animal heart. However, there is still a big gap with mapping the patient's cardiac system, and the next research goal could be to construct complete live animal models of arrhythmias for UCSDI testing.

## Discussion

The current AE effect-based source imaging method is an emerging non-invasive neuroimaging technique. Recently, an increasing number of studies have focused on this method. As mentioned above, it is a promising method for non-invasive brain and heart examinations due to its high spatiotemporal resolution. Overall, the results so far encourage the current AE effect-based source imaging method as a non-invasive medical diagnostic technique.

However, there are some potential problems when applying this technique to clinical applications in neuroimaging. (1) Many experiments are still based on saline to acquire AE signals, and such conditions are too ideal, ignoring the inhomogeneity of real physiological tissue conductivity and sensitivity distribution. Therefore, it is crucial to configure non-homogeneous phantoms and construct more realistic physiological models using *in vitro* biological tissues and live animals. Furthermore, for the setup of simulated currents, most of the current studies have implanted electrodes to construct dipole design experiments. However, this experimental approach ignores many realistic physiological factors of living tissues such as electrophysiological signal strength, switching currents, and electrode contact. These physiological factors need to be further investigated before current source imaging techniques can be applied in the clinic.

(2) Most reported current source imaging methods use the gradient and Laplacian of the AE signal to approximate the current density field and current source distribution, respectively. Although previous studies have shown that this approximation is reasonably accurate if the target current source distribution can be modeled as a dipole source. In future imaging studies of 3D bulk conductors, a more general 3D inverse solution of the AE equation is needed. The inverse solution of this equation is a direct mapping to a real current source. In addition, since the quality of AE signal directly determines the imaging quality, but its amplitude is low and easily masked by noise. Therefore, efficient high-frequency filtering methods and high SNR AE signal decoding algorithms for different environments and experimental conditions also need to be further investigated.

(3) Image acquisition speed is limited by the mechanical scanning of the unit ultrasound transducer. In theory, current source imaging methods such as AEI can be as fast or faster than conventional PE ultrasound. Because it relies only on the unidirectional propagation of acoustic pulses, it may be able to achieve twice the frame rate of typical ultrasound systems, which may provide valuable feedback in the treatment of cardiac and neurological diseases. For future real-time 3D or 4D AEI, for example, it will be necessary to integrate an array with advanced beamforming technology on the platform.

(4) The resolution and sensitivity of this imaging method are still determined by the size of the ultrasound focus and the amplitude of the current gradient, respectively. However, during ultrasound transcranial treatment, the skull causes strong distortions in phase and amplitude. The distortion of the post-transcranial acoustic field will greatly affect the accuracy of imaging. In addition, higher ultrasound frequencies do not penetrate as deeply as lower ultrasound frequencies, but have higher resolution. In future research, the development of phase-controlled transducer technology must be advanced simultaneously. It can compensate for the acoustic beam propagation distortion caused by the skull and improve the imaging accuracy.

In future studies, we can further combine AE imaging with multisensory stimulation. Studies have shown that cortical areas such as the posterior parietal cortex (PPC), premotor cortex and posterior superior temporal sulcus (pSTS) are involved in multisensory information processing. Several subcortical structures such as the superior colliculus, amygdala and thalamus also function in multisensory information processing (Ghazanfar and Schroeder, 2006). It is important to note that the prefrontal cortex (PFC) is recognized as the higher cortex of the brain, receiving projections from multiple cortices including visual, auditory, olfactory, and somatosensory (Van Eden et al., 1992). Each of these different sensory stimuli generates evoked potential (EP) in the corresponding brain regions. We can use FUS to periodically modulate evoked potentials in target brain regions (e.g. visual evoked potential (VEP) (Di Russo et al., 2002; Kodama et al., 2010; Luo et al., 2015), auditory evoked potentials (AEP) (Picton et al., 1974; Daniels et al., 2018; Rieger et al., 2018), somatosensory evoked potential (SEP) (Fedele et al., 2017; Fredland et al., 2019; Insola et al., 2019), etc.) and further realize high spatiotemporal resolution bio-current source imaging based on AE effect.

## Conclusion

The paper summarizes the results of the literature on acoustoelectric effect and its biological current source imaging in the past decade. The research progress, existing problems and future directions of this imaging technique in brain neurons, guided brain therapy and heart are discussed. In conclusion, the inverse solution of the AE equation needs to be further investigated and the FUS stimulation parameters and targeting accuracy need to be optimized with additional consideration of the real physiological factors of the tissue. There is no doubt that the AE effect-based biological current source imaging method is a very promising technique that has the potential to become one of the effective tools in neuroimaging.

## Data availability statement

The original contributions presented in the study are included in the article/supplementary material, further inquiries can be directed to the corresponding author/s.

## Author contributions

HZ and MX designed the study and wrote the manuscript. ML, XS, and FH collected the relevant literatures. SC and DM reviewed and edited the manuscript. All authors read and approved the submitted manuscript.

## Funding

This work was supported by the National Key Research and Development Program of China (No. 2017YFB1300302), the National Natural Science Foundation of China (Nos. 81630051, 61976152, and 81801787), and the Young Elite Scientist Sponsorship Program by CAST (2018QNRC001).

## Conflict of interest

The authors declare that the research was conducted in the absence of any commercial or financial relationships that could be construed as a potential conflict of interest.

## Publisher's note

All claims expressed in this article are solely those of the authors and do not necessarily represent those of their affiliated organizations, or those of the publisher, the editors and the reviewers. Any product that may be evaluated in this article, or claim that may be made by its manufacturer, is not guaranteed or endorsed by the publisher.
